# Medico-Legal Cases

**Published:** 1907-04-13

**Authors:** 


### MEDICO-LEGAL CASES.

### PROFESSIONAL CONFIDENCES OF MEDICAL MEN IN A COURT OF LAW.

The leading authority upon this subject is the well-known decision of Lord Mansfield in the famous trial for bigamy of *the Duchess of Kingston* in 1776 (20 St. Tr. 572). In that case Cæsar Hawkins, serjeant-surgeon to the King, when called for the prosecution, objected to give evidence as to anything that had come before him confidentially in his capacity as a surgeon. Lord Mansfield then said : "A surgeon has no privilege to avoid giving evidence in a court of justice, but is bound by the law of the land to do it. . . . A surgeon has no privilege, where it is a material question in a civil or criminal cause to know whether parties were married, or whether a child was born, to say that his introduction to the parties was in the course of his profession, and in that way he came to the knowledge of it. I take it for granted that if Mr. Hawkins understands that, it is a satisfaction to him, and a clear justification to all the world. If a surgeon was voluntarily to reveal these secrets, to be sure he would be guilty of a breach of honour, and of great indiscretion; but to give that information in a court of justice, which by the law of the land he is bound to do, will never be imputed to him as any indiscretion whatever." It will be seen that Lord Mansfield makes a distinction between disclosures in a court of justice under the sanction of the law and the breach of professional confidence under other circumstances. What was then laid down as the law has been so regarded ever since.

In *Wilson* v. *Rastall* (4 T.R. 759), which related to the evidence of an attorney, Mr. Justice Buller said : "The privilege is confined to the cases of counsel, solicitor, and attorney; but in order to raise the privilege it must be proved that the information which the adverse party wishes to learn was communicated to the witness in one of those characters; for if he be employed merely as a steward he may be examined. It is indeed hard in many cases to compel a friend to disclose a confidential conversation; and I should be glad if by law such evidence could be excluded. It is a subject of just indignation where persons are anxious to reveal what has been communicated to them in a confidential manner. I take the distinction to be now well settled, that the privilege extends to those three enumerated cases at all times, but that it is confined to these cases only. There are cases to which it is much to be lamented that the law of privilege is not extended; those in which medical persons are obliged to disclose the information which they acquire by attending in their professional characters."

In *Rex* v. *Gibbons* (1 C. and P. 97) the prisoner was indicted for the murder of her bastard child. Mr. Cozens, a surgeon, was called to prove certain confessions made by the prisoner to him. The witness objected to give such evidence, on the ground that, at the time of the statement, he was attending the prisoner in the capacity of a surgeon. Mr. Justice Park said : "That is no sufficient reason to prevent a disclosure for the purposes of justice." The witness also stated that he had held out no threat or promise to induce her to confess; but a woman who was present said that she had told the prisoner she had better tell all, and then the prisoner made certain confessions to the witness. Mr. Justice Park, after consulting with Baron Hullock, laid down that, as no inducement had been held out by Mr. Cozens, to whom the confession was made, and the only inducement had been held out (as was alleged) by a person having no sort of authority, it must be presumed that the confession to Mr. Cozens was a free and voluntary confession. If the promise had been held out by any person having any office or authority, as the prosecutor, constable, &c., the case would be different.

In *Greenough* v. *Gaskell* (Myl. and K. 98) Lord Brougham agreed with this view while speaking of the privilege in the case of legal advisers, though he added that "certainly it may not be very easy to discover why a like privilege has been refused to others, and especially to medical advisers."

In *Wheeler* v. *Le Marchant* (17 Ch.D. 681) Lord Justice Jessel said : "The principle protecting confidential communications is of a very limited character. It does not protect all confidential communications which a man must necessarily make in order to obtain advice, even when needed for the protection of his life, or of his honour, or of his fortune. There are many communications which, though absolutely necessary because without them the ordinary business of life cannot be carried on, still are not privileged. The communications made to a medical man whose advice is sought by a patient with respect to the probable origin of the disease as to which he is consulted, and which must necessarily be made in order to enable the medical man to advise or to prescribe for the patient, are not protected. Communications made to a priest in his confessional, on matters considered by the penitent to be more important even than his life or his fortune, are not protected. Communications made to a friend with respect to matters of the most delicate nature, on which advice is sought with respect to a man's honour, or reputation, are not protected. Therefore it must not be supposed that there is any principle which says that every confidential communication which it is necessary to make in order to carry on the ordinary business of life is protected."

The reason why the evidence of medical men is not privileged is perhaps not difficult to see. Lord Brougham, in *Greenough* v. *Gaskell*, gives as the reason for the privilege of legal advisers that it is out of regard to the interests of justice, which cannot be upheld, and to the administration of justice which cannot go on, without the aid of men skilled in jurisprudence, in the practice of the courts, and in those matters affecting rights and obligations which form the subject of all judicial proceedings. If the privilege did not exist at all, everyone would be thrown upon his own legal resources; deprived of all professional assistance, a man would not venture to consult or tell his counsellor half his case. Medical men, on the other hand, have nothing to do, as such, with the administration of justice. The reason of the privilege in relation to legal advisers does not extend to them. It is not absolutely necessary in the interests of justice that inviolable secrecy should attach to all transactions between a man and his medical adviser. The reason for the obligation of honour on the part of medical advisers is not to enable the patients by whom they are consulted to obtain justice according to law, but to secure that privacy in personal matters which every man is lawfully entitled to expect.

